# A call for biological data mining approaches in epidemiology

**DOI:** 10.1186/s13040-015-0079-8

**Published:** 2016-01-04

**Authors:** Shannon M. Lynch, Jason H. Moore

**Affiliations:** Cancer Prevention and Control, Fox Chase Cancer Center, Philadelphia, PA 19111 USA; Department of Biostatistics and Epidemiology, Institute for Biomedical Informatics, Perelman School of Medicine, University of Pennsylvania, Philadelphia, PA 19104-6021 USA

Forging a partnership between the traditionally distinct disciplines of informatics and epidemiology is becoming increasingly necessary. Epidemiology is the study of the distribution and determinants of disease. Traditionally, epidemiology has focused on univariate analysis and studied single or a small number of risk determinants and their relationship to health outcomes. However, given the multifactorial and complex nature of chronic diseases, such as cancer, epidemiology has shifted its focus from single risk factors to multilevel conceptual frameworks of health that serve to integrate and study multiple risk factors and how they interact across 3 main levels: 1) the macro-environment, defined by factors outside an individual, such as where a person lives, their family/social circumstances , and environmental exposures; 2) the individual, which includes behaviors, such as smoking, and psychosocial factors; 3) biology, which includes the study of genes and other biomarkers [1]. Similar to the biologic concept of epistasis, understanding which risk factors are most relevant to disease and their interactions is exceedingly convoluted within one level, let alone across multiple levels. Further, few existing population and clinic-based study samples include risk factor information at each of these levels. Thus, it is difficult to test these conceptual frameworks from both a data availability and analytic standpoint.

Epidemiology could benefit from entering the “big data” arena and has begun to do so with studies at the biologic level. Advances in –omic technologies have led to the generation of large datasets in genomics and proteomics; however, publically available datasets that contain risk factor information at both the individual and macro-environmental level remain untapped and underutilized. For instance, U.S. Census and U.S. Consumer Spending data could be combined with existing clinical biorepositories and linked through a geocode to test hypotheses related to the interaction of the macro-environment and biology in disease etiology and prognosis. A recent report of emerging macrotrends in Epidemiology suggests that data integration and generation of large social, environmental, and clinical datasets should be a core competency in epidemiologic training [2]. However, the creation of these enormous datasets is futile without the ability to analyze and manipulate big data.

Analyzing big data requires knowledge and execution of data mining techniques. Like most biomedical sciences, epidemiology relies heavily on reductionist approaches that use standard regression models (i.e. linear, logistic, multilevel) based on statistical assumptions that may not reflect the true nature of how a risk factor or group of risk factors influence disease etiology and prognosis. For example, genome-wide association studies (GWAS) have yielded new insights into disease processes, but have proven to have little prognostic value, perhaps due to a stringent emphasis on identifying true positives, as well as a focus on the analysis of univariate as opposed to joint effects [3]. Complex Systems approaches and agent-based modeling (ABM) have become increasing popular in epidemiologic investigations, given their focus on interactions or joint effects. ABM is a type of systems algorithmic approach that accounts for the recognition of feedback, interference, change over time, and nonlinearities among risk factors *a priori* [4], based on existing knowledge and observation, but it is not a true data mining technique that can identify novel risk factors or groups of risk factors empirically.

Epidemiology is in need of more powerful modeling approaches that relax model assumptions and allow for more empiric investigations of large scale, joint biologic, social, and genetic datasets. Biological data mining approaches, particularly those related to artificial intelligence and machine learning, could address current epidemiologic limitations and are starting to be explored in population-based studies that include patient and biologic level data [5, 6]. These approaches are model-free, nonparametric, and allow for high performance computing that can incorporate artificial intelligence approaches with human knowledge [6]. Some machine learning approaches, such as neural networks [6] and learning classifier systems [7], have demonstrated an added statistical benefit, as well as revealed effects missed by traditional regression frameworks [3]. While one of the limitations of machine learning algorithms has been validation and interpretation of findings, epidemiology often plays an important role in evaluating inferential statistical methods [8]. Thus, the computational capacity offered by machine learning algorithms, which can allow for the identification of complex interactions across multiple data levels and multiple risk factors, warrants further study in epidemiologic investigations.

Epidemiology and informatics can be linked through common data mining methods applied across macro-environmental, individual, and biologic data sources. A partnership with epidemiology would expand the application and reach of data mining methods beyond just genomic or proteomic investigations. Applying big data approaches, namely the creation of large scale datasets from existing resources, as well as data mining methods (i.e. those related to machine learning), to test hypotheses related to epidemiologic, multilevel conceptual models will likely have implications for improving understanding of disease etiology and prognosis. Informatics can aid in methods development and epidemiology can assess the precision, accuracy, and effectiveness of inferences made using big data approaches [8]. Thus, an Epidemiology-Big Data collaboration is of mutual benefit to both groups, and it is the goal of *BioData Mining* to foster these type of collaborations.
